# A scoping review of ethical aspects of public-private partnerships in digital health

**DOI:** 10.1038/s41746-025-01515-3

**Published:** 2025-02-27

**Authors:** Marieke A. R. Bak, Daan Horbach, Alena Buyx, Stuart McLennan

**Affiliations:** 1 Institute of History and Ethics in Medicine, Department of Preclinical Medicine, TUM School of Medicine and Health, Technical University of Munich, Munich, Germany; 2 Department of Ethics, Law and Humanities, Amsterdam UMC, University of Amsterdam, Amsterdam, The Netherlands; 3 Institute for Biomedical Ethics, University of Basel, Basel, Switzerland

**Keywords:** Ethics, Society, Health policy, Medical ethics

## Abstract

Partnerships between public and private organizations in digital health can promote more accessible, affordable, and high-quality care, but they also raise ethical and governance challenges. We searched PubMed, EMBASE, and Web of Science, identifying 46 studies examining ethical aspects of digital health public-private partnerships (PPPs). Three key themes emerged: data privacy and consent, ensuring public benefit and access, and good governance and demonstrating trustworthiness. We provide recommendations for each theme. To foster responsible innovation, we conclude that early and contextual operationalisation of ethics guidelines in PPPs is necessary to balance respect for fundamental values with the pursuit of impactful innovation. If PPPs become more successful as a result, this contributes to reducing the research waste of failed collaborations. Further research should clarify the scope of PPPs and definition of ‘public benefit’, and we call for critical study on the ‘economization’ of digital health promoted by public and private sector organizations.

## Introduction

Public-private partnerships (PPPs) exemplify ‘open innovation’ networks where data, expertise, and risk are shared for the mutual benefit of public and private partners^1,2^. Such partnerships are grounded in common interests and joint decision-making, and the World Bank defines a PPP as a “long-term contract between a private party and a government agency, for providing a public asset or service, in which the private party bears significant risk and management responsibility” (2012, p.11)^3^. PPPs are stimulated in the developing field of ‘digital health’, predicated on the idea that both public and private partners are needed to develop accessible, affordable and high quality health technology. The European Commission unites public organizations and private commercial entities in the Horizon Europe research funding framework^4^, and the government of the Netherlands has, for example, established the Health Holland programme to facilitate and finance PPPs for healthcare innovation^5^. The curation of large patient cohorts and disease registries by governments and academic hospitals makes them desirable collaboration partners for technology companies developing data-driven tools^6^. While some digital health PPPs are based merely on sharing health data between public and private organisations out of a common interest (i.e. ‘data partnerships’^7^), others truly pool their resources or expertise for the purpose of jointly providing care or developing innovations in more long-term collaborations (i.e. ‘strategic partnerships’^8^).

Although the rise of digital health PPPs provides opportunities for societal impact, they also raise important ethical issues for those people whose data informs digital health technologies (mainly patients and citizens), future users (including clinicians), and for society as a whole. People are reluctant to share health data with private companies, so solidarity and trust in medical science can be affected by PPPs^9–11^. This reluctance stems from growing power imbalances between tech companies and data subjects in the age of ‘surveillance capitalism’^12^, which refers to an economic system where financial profit may be obtained from the systematic collection of personal data to predict and influence consumer behaviour. Commercial interests can also cause a lack of transparency about data protection issues, and the obscurity of commercial artificial intelligence (AI) models limits their governance and the assessment of bias^13^. Whenever commercial companies participate in medical research and innovation, there is the threat of conflicts of interest related to financial gains and publications^14^, and of restricting access to publicly funded datasets and applications in order to serve business interests^15,16^. In healthcare, these trends were exemplified by Google DeepMind’s taking of NHS patient records without the consent of those affected^17^. This case is described in Box 1, along with two other key illustrations of controversial digital health PPPs. Of note is that the ethics discourse is focused mainly on companies’ commercial interests, while public institutions and researchers may also have certain interests that conflict with those of patients and citizens, as we will discuss further on in this article.

**Fig. 1:**
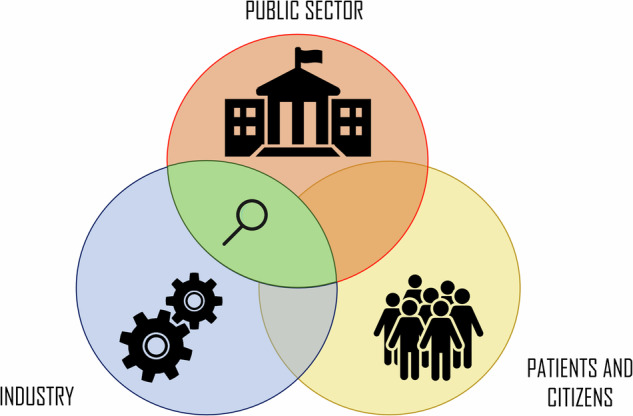
Partnering spaces in digital health. This review focuses on the intersection between the public sector and industry and, in principle, this also includes partnerships where patient organisations or civil society are involved as additional partner. Based on the theoretical framework by van Tulder and Pfisterer^77^.

With the rise of public-private collaboration, it becomes imperative that appropriate policies are created to “avoid socialization of risk and privatization of rewards”^18^ in digital health research and innovation. However, data protection laws are necessarily general and there are currently no specific (inter-)national ethics guidelines specifically for digital health PPPs. Although previously there has been a lot of focus on partnerships with the pharmaceutical sector, such partnerships have important differences from those with digital technology companies; *big pharma* has long time experience with human subjects research and oversight by Institutional Review Boards, while *big tech* has operated largely outside the regulatory constraints of medicine^19^. Thus, we cannot simply copy lessons from pharmaceutical PPPs one-to-one. The aim of this review, therefore, is to systematically collect and review the ethical issues that are raised in digital health PPPs. This will provide a starting point for further research and practical ethical guidelines. In our analysis, we used the World Bank definition of PPPs but expanded the focus from government agencies to the entire public sector including (academic) hospitals, and in principle also included within our scope any papers discussing tripartite partnerships wherein civil society functions as an additional third partner in a PPP (Fig. 1). Regarding the latter, we find such partnerships to be within the scope of PPPs but note that in reality patients and citizens have few formal opportunities to have their voices heard, as this wider range of stakeholders is often excluded from digital health partnerships.

Box 1 Key examples of controversial PPPs in digital health ***Care.data***

The care.data programme was proposed in the UK in 2013 to collect and share anonymised patient data from general practitioner (GP) records with the National Health Service (NHS) and other organizations for research and planning purposes. However, concerns arose quickly, initially focusing on the information leaflets that looked like junk mail and did not include opt-out options. Critics argued that the scheme lacked transparency, proper consent procedures, and safeguards to protect sensitive data from being accessed or exploited by third parties, including private companies. Amidst public outcry and criticisms from medical professionals, the care.data programme faced significant delays and ultimately was abandoned in 2016.

***NHS / DeepMind***

In 2015, the NHS partnered with DeepMind, a Google-owned AI firm, to develop "Streams", an app that would help healthcare professionals with detecting and managing acute kidney injury. DeepMind accessed 1.6 million patient records from Royal Free London hospital without patient consent, raising concerns about privacy, oversight, and transparency of the collaboration between a tech giant and public organisation. The UK’s Information Commissioner’s Office found the data-sharing agreement violated data protection laws. In the aftermath of the controversy, DeepMind and the NHS revised their agreements.

***State of Iceland / deCODE***

In 1998, Iceland granted deCODE Genetics exclusive rights to create a national database combining medical and genetic records. The project lacked transparency and proper consent procedures, and critics feared that the genetic information could be exploited for profit by deCODE Genetics or shared with third parties without consent. In response to international controversy, the legislation was withdrawn and the national database failed, but deCODE Genetics revised its consent procedures to improve information provision and give Icelandic citizens the option to opt out of the adapted project now led by deCODE.

## Results

### Included studies

**Fig. 2:**
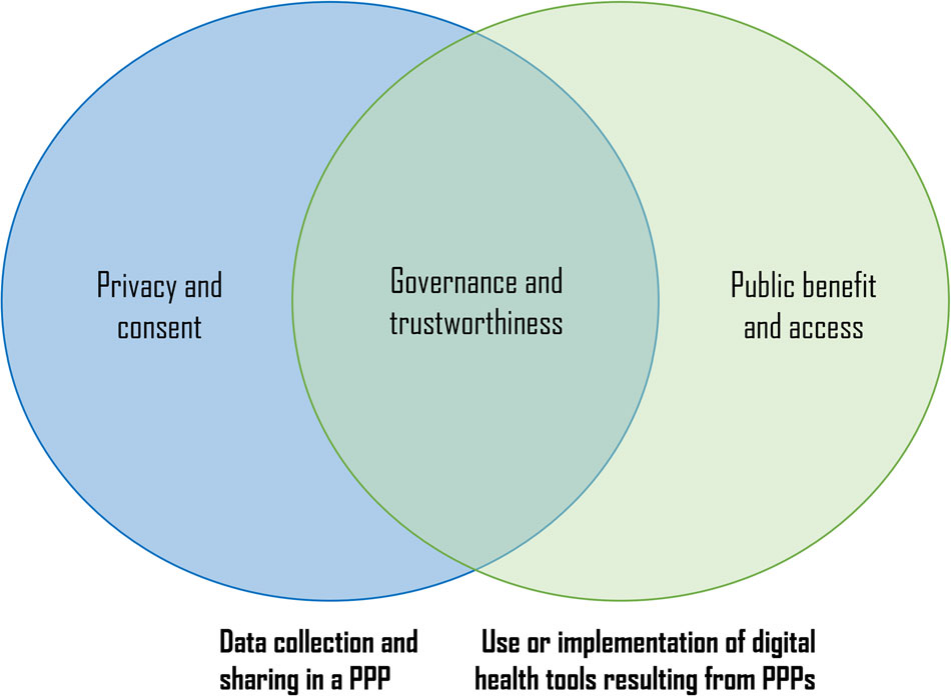
Key ethical themes in digital health PPPs. Overview of the main ethical themes distributed over the two general phases of digital health technology development, i.e. the data collection and analysis phase, and the implementation phase.

A total of 46 studies were included in this review (Supplementary Table 1: screening and inclusion flowchart, Supplementary Table 2: characteristics of included studies). Of these, 82.6% (38/46) were published in the last five years. The majority of papers (56.5%; 26/46) described European PPPs: in Denmark, Finland, Iceland, Italy, Switzerland, United Kingdom (UK), or Europe generally. Other papers (17.4%; 8/46) referred to the United States (*n* = 4), Canada (*n* = 1), Australia (*n* = 1), Israel (*n* = 1), and Singapore (*n* = 1). The remaining 12 (26.1%; 12/46) papers contained general ethical discussions without reference to a specific country context. Five specific cases of digital health PPPs were analysed in detail in more than one paper: the care.data scheme in the UK (*n* = 6), the partnership between the UK’s National Health Service (NHS) and Google’s DeepMind (*n* = 6), the collaboration between the State of Iceland and deCODE Genetics (*n* = 4), the Findata programme in Finland (*n* = 3), and the Google/Apple collaboration with national governments during the COVID-19 pandemic (*n* = 3). Most included studies (*n* = 30) discussed ‘data partnerships’, whereas fewer studies (*n* = 16) analysed ‘strategic partnerships’. No article discussed tripartite partnerships in which patient organizations or citizen groups join the public-private collaboration as a third partner. Three overarching ethical issues throughout the lifecycle of digital health PPPs were identified: data privacy and consent, public benefit and access, and trust and governance (Fig. 2). In Table 1, we provide an overview of key recommendations for each theme, derived from the literature, to support the creation of ethically informed PPPs in digital health. These recommendations should be viewed in conjunction with each other because, for instance, individual consent is not by itself a sufficient safeguard for privacy and it does not protect against broader societal harm.

**Table 1:** Recommendations for ethically informed digital health PPPs, synthesized from the reviewed literature

Privacy and consent for data processing in PPPs
1 PPPs should be aware that privacy concerns can be targeted both at the private and the public partner.
2 Rules for sharing data within PPPs should not focus only on identifiable data, as this neglects broader issues of trust and fairness that remain when data is deidentified.
3 Opt-in consent should be the standard when data is shared in PPPs for purposes outside of direct patient care—unless there are good reasons for a clear and transparent opt-out model, backed by public and patient engagement efforts.
4 Re-consent or notification is needed in case data are transferred (e.g. after bankruptcy) or when new uses arise.

Public benefit and access to the results of PPPs
5 The potential value contribution of PPPs should be recognised, and secondary commercial profit can be accepted when public benefit is achieved too.
6 PPPs should explicate the type of benefits of the proposed partnership early on: context-sensitive guidance is needed on whether benefits outside of healthcare are acceptable grounds for starting a PPP.
7 PPPs may only appeal to public benefits to override safeguards like consent when this is proportional and transparent.
8 The rhetoric of (data) solidarity should not be used to manipulate people into sharing their data with PPPs: this is problematic because beneficiaries are generally unclear and scientific advancements uncertain.
9 PPPs should define IP rights and pricing upfront. Publicly collected data should remain a public good, and public healthcare systems should have free access to goods and services developed in PPPs.
10 Monopolization should be avoided by not granting a single company exclusive access to data, and more research is needed on the negotiating power of the public partner in PPPs.

Good governance and demonstrating trustworthiness of PPPs
11 Public acceptance of and internal trust within PPPs require good and transparent information provision and demonstrably trustworthy partners: a sense of urgency and efficiency should not be used to override appropriate governance efforts.
12 Rather than owning or even selling data, the public partner should be a data steward who protects and promotes the use of data in PPPs, based on clear policies.
13 PPPs should be overseen by ethics boards with sufficient distance from the commercial partner and with expertise in digital health, which may require additional training.
14 PPPs should be transparent about their aims, performance indicators, contractual agreements, and results, and partners should be joint data controllers when this is appropriate.
15 Rather than mere awareness-raising, PPPs should involve meaningful engagement with public and patient representatives.

### Privacy and consent for data processing in PPPs

The literature describes privacy concerns and uncertainties around the scope of regulations in relation to digital health PPPs. Although PPPs are started from some common interest, private and public institutions may have different secondary goals that conflict with privacy^20,21^. Various authors frame the private sector as ‘tyrannical’ and argue that digital health PPPs are facilitating sphere transgressions of internet companies into the sphere of health, which violates the public’s expectations about privacy^22–24^. However, privacy concerns can be targeted at both the private *and* the public sector, focusing respectively on risks of commercialization (e.g. unwanted profiling, or an increase in inequalities) and state surveillance (e.g. misuse by law enforcement agencies, or the normalization of pandemic surveillance)^25,26^. Data sharing in PPPs can give rise to ‘group privacy’ concerns related to broader harms around stigmatisation or increasing inequality, as well as to concerns about individual re-identification, which may be particularly easy in smaller countries like Finland or Iceland that are ideal ‘population laboratories’ for health and genetic research^27^. While data protection regulations are aimed at safeguarding data privacy, they do not always cover data processing in PPPs. Foreign companies may be based outside the jurisdiction of the data subjects; moreover, many regulations only apply to medical records in public organisations and not to commercial health data collected in consumer apps or wearables^28–30^. In addition, most data protection laws only cover data from identifiable subjects, but some people have principled objections to secondary commercial uses even when their data is successfully de-identified, so current data protection regulation may not suffice for digital health PPPs^30,31^.

Another key topic in the literature was informed consent for data sharing in PPPs. In cases of sharing publicly stored health data with companies, the process of giving informed consent is increasingly framed as an individual trade-off between exchanging ‘your’ personal health data for ‘public benefit’. Indeed, according to most data protection and health laws, individual consent might not be strictly required if there is a clear and important public interest. However, Vezyridis and Timmons discuss how this binary view disregards that public health innovation is only possible because of long-standing individual rights^24^. Moreover, various authors note that making such a trade-off or cost-benefit calculus is not truly possible because of patients’ inability to adequately control their data and because consent is an insufficient safeguard for broader societal harms^32,33^. Still, consent is seen as valuable for purposes of transparency and for giving people the option to *veto* certain uses of their data, as well as for maintaining trust in health research. Various major collaborations with industry were unsuccessful because data subjects´ consent was not adequately obtained and trust was harmed as a result. In addition to the care.data^24,30,34–37^, DeepMind^30,38–42^ and deCODE^30,43–45^ controversies presented in Box 1, the literature describes criticism of Finland’s data infrastructure which does not allow opting out of data sales to commercial entities^46^, while in Italy, the unconsented transfer of health data from the government to IBM Watson Health was blocked due to legal uncertainties^41^. In the US and UK, identifiable data sharing without consent is allowed when companies provide services for patient care, and there are debates whether this exception has been misused in data sharing PPPs between healthcare organisations and Google, where a ‘direct care’ relationship did not truly exist or at least not for all data subjects^28,38,39,42^.

A number of scholars have argued that some form of consent should be required for sharing health data with commercial companies, even though the risk of selection bias inherent to the consent process may be exacerbated when data subjects have to specifically consent for commercial use^20,28,47^. Whether via an opt-in or opt-out process, these authors find that data subjects should be informed of how and for how long their data will be used in the PPP. Kaplan notes that at a minimum, data subjects should be able to opt out of commercial data use^48^. According to the reviewed literature, such opting-out should be easy and good reasons (i.e. those generally found relevant and fair) for accepting data sharing in PPPs must be specified in consent documents^31^, and the provided information should be kept up to date, e.g. by announcing new uses on a public website or through dynamic online consent processes^49,50^. Moreover, examples of bankruptcy of the original data holder such as in the SharDNA case in Italy^50^, show that notice or consent might need to be renewed when data are transferred to another organisation or company.

### Public benefit and access to the results of PPPs

A key ethical requirement for establishing digital health PPPs is that the eventual results or tools benefit the public interest, especially when the data comes from the public sector^20,30,51^. In various legal frameworks such as in the EU, the ‘public interest criterion’ is a potential legal ground for not obtaining consent. We should note here that something which is in the public interest is not necessarily of direct public benefit, but in the reviewed literature the terms ‘benefit’ and ‘interest’ were used interchangeably. In any case, the potential value contribution of digital health PPPs is widely recognised and these initiatives are welcomed by researchers because they address limitations such as a lack of technological infrastructure or expertise in big data analytics^22,35,36,41,49,52^. In other words, “avoiding PPPs altogether would not serve the public interest”^49^. When public benefit is achieved, secondary commercial profit is generally accepted given the obligations that companies have to shareholders, employees and customers^51,53^. The ethics literature highlights different types of public benefits: improvements in direct patient care, advancements in scientific knowledge, cost-savings to the healthcare system, and even benefits to the national economy. Various authors argue that the important distinction is not just between public and private benefit, but also between a plurality of arguments (‘normative repertoires’^23^) that provide legitimacy to a PPP, which range between driving research, to improving healthcare, achieving financial and economic gain, and maybe even by monetising the data as such^25,35,41^. Due the ambiguity around the public interest requirement, it may not restrict data sharing but actually pave the way for expanded uses in the broader public interest, like promoting economic growth through PPPs^32,54^.

Namely, the shift from welfare state data regimes to data-driven health economies can go hand in hand with a process of national ‘solidarization’^46^, whereby citizens are called upon to contribute their data to PPPs in order to benefit the public interest, despite uncertainties about what this interest is and who the actual beneficiaries are, and with the risk that eventually only those who share their data can use health services^27^. The main problem with this solidarization is that especially long-term strategic partnerships are often not aimed at a specific health benefit but rather at improving the research and business climate and at making the country in question a “famous and a leading country in the digital social services and healthcare field”^54^. The increased involvement of private companies can be related to a history of budget cuts and worries about sustainability of healthcare systems^23^. In the example of deCODE, the partnership tapped into citizens’ economic worries: at a time when fish stocks declined, a new source of income could be found in health technology that profited from the genetic homogeneity of the Icelandic population^45^. As such, solidarization is grounded in ‘population branding’—i.e. employing marketing strategies to position a country’s population as special to attract investments in data as a national resource^44^—thereby excluding those people who are not part of the branded population, and it is thus incompatible with a more universal solidarity^46^. Moreover, when the rhetoric of solidarity is used to promote a neoliberal view of health and research, this harms the trust of citizens who wish to “further the common good without being manipulated into doing it”^36^.

In the literature, we found no consensus on which types of benefits are acceptable – i.e. whether PPPs should only be started when they bring improvements to future patients’ health or the healthcare system, or if PPPs that strengthen economic and research environments are also acceptable^29,51,53^. Views about this vary for different cultural (country) contexts and for the different social roles that one individual may have: “what patients care about as patients cannot be equated with what patients care about as citizens who are part of a much wider social endeavour”^34^. Whether specific benefits are seen as acceptable also depends on the level of certainty that these benefits will truly be brought about. Solidarization may not be appropriate also because there is some healthy scepticism about the actual benefits of tools developed in digital health PPPs, as these depend on the quality of the data and research, which should be subject to scientific review^21^. Especially when PPPs use data generated or collected by the commercial partner, the datasets are often patchy and biased because they are self-reported or passively collected by users who use the service (e.g. a smartwatch) inconsistently^22,44,55^. Moreover, the absence of quality meta-data may complicate linkage of data between different partners^31^. Finally, there may be a conflict of interest in the sense of a felt obligation by public researchers to the company that co-funds their studies, potentially leading to distortions in the design or analysis process^21^. Various scholars argue therefore that as a general rule, overriding safeguards like individual consent by appealing to public benefits may be acceptable only when this is proportional and transparent^25,28,32^. Partners should define early on their vision for how their PPP creates public benefit: this vision should be based on performance indicators which are actually tracked, to avoid ‘lip service’ or ‘ethics washing’ when companies or governments merely have an interest in accountability in order to protect their image^49,51^. This would also help avoid strategic (mis)use of the concept of public interest for retaining credibility when expectations based on one particular definition are not met^54^.

Other issues discussed in the literature relate to access and intellectual property. In the most common terms, a public benefit may refer to one that is widely available^56^, which the literature describes is not always the case if private interests lead to digital health products being overpriced or otherwise inaccessible^51^. Various authors raise concerns about the exacerbating of inequalities when benefits are not fairly shared due to commercial interests^28,32,38^. Ideally, according to various authors based on their case studies, the healthcare institution providing health data should have free access to the resulting goods and services^25,36,39,57^ or at least recover the costs of providing the data and knowledge through data access fees^56,58^, but this is not always the case because the ideal of benefiting public healthcare, research and education may clash with the profit-driven aims of companies. It is known that the accessibility of digital health applications depends for a large part on intellectual property (IP) arrangements designed to protect industrial partners’ competitive advantage. Ballantyne and Stewart advise that public and private sector collaborations take the time to identify shared interests and clearly articulate how benefits will be distributed^38^. In the example of the DeepMind controversy, the NHS might have secured joint IP rights with a longer negotiation process involving a broader range of stakeholders^38^. These discussions should cover fair pricing, according to Winkler et al.^51^. Moreover, Landers et al.^49^ argue that the data generated in a PPP should be made open after the project is finished, according to the principles of findable, accessible, interoperable and reusable (FAIR) data and that IP rights may be sought only on outcomes mostly achieved by the private partner.

The specific ways in which IP and other benefits are shared need to be determined case-by-case, but there seems to be consensus in the reviewed literature that public health data itself should always remain a public good, given the public investments in collecting it^23,39,49,51^. Digital health data coming from the public sector’s ‘gold mine’ is increasingly viewed as ‘asset’ or ‘public good’ that can create both social and economic benefit^32,46,59^. However, assigning financial value to data is said to be difficult, as this value is often generated at later steps in the development process of digital health tools, so perhaps public benefits are best understood as indirect societal value rather than direct financial value^49^. Finally, in refs. ^39,51^ it is argued that public organisations should remain gatekeepers and stewards of publicly collected data, even if they do not ‘own’ the data in the proprietary sense. However, fulfilling this role might be difficult due to power dynamics and companies’ monopolies.

The need for public datasets and medical expertise in PPPs may give some negotiation power to the public partner as data stewards^25^. Yet ‘big tech’ companies are bringing expertise increasingly in-house by recruiting from top public organisations, leading to ‘brain drain’ whereby the public healthcare sector is becoming more and more dependent on industry^25,32^. With her frame of the ‘Googlization of health’, Sharon^23^ highlights the risks associated with corporate encroachment into health, politics, and public policy spheres, potentially leading to a crowding out of expertise, growing dependencies and concentration of power, and calls for more bioethics research on these power asymmetries. Large tech companies often promote a neoliberal, techno-optimist narrative focused around the individual as autonomous decision-maker, while ignoring societal concerns like designing inequality into digital health tools^60^. When the wider public is not in a position to benefit from the value of their data this can be seen as exploitation or even ‘tyranny’^23,32^. Lanzing et al.^61^ note that it may be more appropriate to speak of a ‘push and pull’ power play between industry and the public sector regulating it, and that values like digital sovereignty are important for the public sector to keep some of this power.

All in all, the reviewed literature shows that while privacy protection is important, a focus on privacy should not draw attention away from safeguarding public benefit of PPPs by being attentive to larger societal risks of diminished power, expertise and access. While power issues play a role in any collaborations with industry (e.g. with pharmaceutical companies funding clinical trials), the impact might be great in the newer and less developed and regulated field of digital health^21^. There also exists a large digital divide because usually only large tech companies have the funds and infrastructure to move into the sphere of digital health^32^, but a fair playing field should be created by providing companies non-exclusive access to data^51^. It is in the public interest to avoid monopolization of large tech companies and to promote competition so that no single actor can control scientific outputs and the pricing of digital health tools^22,40,41,44^.

### Good governance and demonstrating trustworthiness of PPPs

Instances where the consent process is inadequate and people discover commercial involvement after the fact (Box 1), cause an erosion of trust in both the public and private partner. Carter et al.^34^ have analysed this as the failure of obtaining a “social license to operate”, referring to the fact that not only legal requirements should be fulfilled but PPPs should also be sensitive to societal expectations about their conduct. The literature describes how citizens may be unfamiliar with the unexpected secondary goal of many PPPs to contribute not only to public health but also to national or regional wealth: trust in public organisations may come under more pressure as governments increasingly have economic, wealth-related and reputational reasons for engaging in PPPs for digital health. With the rise of PPPs it also becomes unclear whom data subjects should trust: the public or private organisation, the actors within these organisations, or the collaboration as such? Existing relations are changed when healthcare organisations enter into (non-transparent) collaborations with digital technology companies, which can be seen as context transgressions resulting in a trust gap^22,49,57^. Eroded trust in healthcare institutions can impact directly on the patient-physician relationship. In the Google-Ascension collaboration, for instance, clinicians were not informed and thus could not tell their patients about this data sharing partnership, which jeopardises patient trust in the confidentiality of the information shared with their doctor^28^. Even data controllers themselves may have trust issues and reputational concerns when they need to handle data access requests from the ‘other’ sector, so demonstrating trustworthiness is important between partners as well^31,52^.

Some authors state that newly set up digital health PPPs should put in the work to earn and maintain public trust^20,31^, while others think that public sector relations with companies should not be based on trust, which is grounded in uncertainty, but rather on the confidence that they are indeed processing health data in acceptable ways^62^. In order to merit the public’s and each other’s trust, both private and public organisations should first ensure being trustworthy when it comes to handling patient data and reaping benefits from this^37^. In particular, several papers highlight the concern that the rapid pace of deal-making, driven by Big Tech’s ‘move fast and break things’ approach, may ultimately break patient and public trust by compromising governance^29,30,37,39,55^.

Promoting trustworthiness of PPPs can be done by basing their governance on the concept of *‘stewardship’*. The idea of stewardship is less complex to apply than ownership, and more appropriate due to its focus on responsibilities related to data protection, appropriate use, and promotion of public benefit^27,31,38,58^. While a ‘data controller’ already has a legal responsibility to ensure compliance with relevant laws on data protection, the concept of a ‘data steward’ is more encompassing, entailing not merely the protection of participants but also the promotion of research when it fulfils a public need, and the balancing between these two^31^. Despite worries that some governments may themselves facilitate sale of data for secondary use as an economically driven data broker rather than a data steward safeguarding the public interest^44^, various authors state that the public partner should always remain the steward of health data who determines what is appropriate access and use and how privacy should be protected and trust promoted^32,38^.

The literature suggests that an internal PPP committee governs day-to-day access and management issues (such internal committees might be financed through data access fees^20^), based on publicly accessible policies, while external ethical and scientific boards are responsible for oversight^38,50^. Good governance or stewardship requires consulting with these oversight bodies, such as data protection authorities, data protection officers, and institutional ethics committees^56,58,63^. Yet ethics committees for overseeing digital health research are less established than for clinical trials and may lack specific epistemic and ethical expertise, ranging from negotiation skills to knowledge of programming, which highlights the need for guidelines and training of governance and ethics committee members on the ethics of digital health in general and of PPPs specifically^21,25,43,47^. Drafting clear policies and guidelines for specific PPPs is helpful when existing (national) regulation does not suffice, e.g. as it does not cover deidentified data^64^, and such guidance can be further refined through multi-stakeholder consultation including both private companies and public entities^49^.

However, there seems to be no consensus on how strictly PPPs should be regulated and whether there is room for self-regulation by the private partner. Some authors advocate strict penalties for misuse of data, e.g. fines or exclusion from future data access, which may serve as an incentive for corporate responsibility^35,58^, and the enforcement of sanctions was seen by citizen juries as promoting trust in digital health PPPs^30,56^. In the context of fair pricing of the digital health tools, however, Winkler et al.^51^ advise against such penalties as they find that “public moral pressure or fear of reputational damage” would be sufficiently motivating for industry to self-regulate to ensure equitable access. Whether self-regulation is sufficient, remains to be seen. For instance, Powles & Hodson^39^ criticized DeepMind’s self-appointed (and later abolished) ‘independent board’ for hindering true oversight, a concern echoed about the company’s ‘Ethics and Society Fellows’^42^.

The reviewed articles highlight that good stewardship requires particular attention for transparency and accountability of researchers and data stewards, not merely as procedural formality but as fundamental principles that should underpin digital health PPPs, especially when these collaborations are (indirectly) funded by public taxes and use publicly collected data. Consensus in the literature is that transparency constitutes a minimal requirement: people should at the very least be made aware that and how their data are processed in PPPs^30,49,56,64,65^. The public generally has less trust in industry than in the public sector^32,38^, yet in a study of two Australian “citizen juries” who discussed the sharing of data with private partners, those juries did not reject all involvement of commercial companies and understood the need for PPPs in specific cases after explanation^56^. Even though transparency and other demonstrations of trustworthiness are no guarantee for eliciting trust, there is an intrinsic link between transparency and the social license, because transparency to patients, employees and the public helps facilitate public debate^38^. Transparency is important at different levels, ensuring that datasets and algorithms are transparent but also in terms of disclosing potential conflicts of interest (COIs) and being transparent about the (commercial) purposes and practices of data sharing as laid out in agreements^21,58^. The literature also suggests several accountability requirements: namely, key performance indicators should be described and publicly monitored^49^, and clear lines of responsibility and effective accountability mechanisms are needed, coupled with proactive stakeholders engagement, and possibly with compensation measures for demonstrable harm caused by data sharing in PPPs^31,51^.

Yet the commercial and reputational concerns of private and public partners can impede these efforts—especially since private companies are not bound to public transparency and accountability in the way that the public sector is, which can lead to power asymmetries and create conflict between commercial secrecy and public oversight mechanisms^26,49^. Various case studies underscore the challenges in balancing transparency with commercial interests, with lack of clarity in contractual arrangements and data governance frameworks raising concerns about data sovereignty and public trust^36,37,39,49^. In the DeepMind controversy, one accountability issue was that the company was labelled only as a data processor rather than joint data controller, despite having significant control over data use^39^. Similarly, Google’s partnership with Duke University positioned the university as “assisting” Google, instead of the other way around, showing the limited decision-making power of the university^22^.

Finally, taking transparency and accountability a step further, the reviewed articles underscore the critical role of public engagement in navigating the complexities of PPPs. This entails organising ongoing dialogue to address concerns and garner support from stakeholders, especially the communities whose data are shared. In addition to controversial PPPs that did not consult stakeholders, there are various ‘best practice’ cases of achieving community buy-in by setting up patient and public involvement (PPI) strategies early on. For instance, Piciocchi et al.^50^ describe two cases where early engagement with local communities through town hall meetings and constant communication fostered trust and high participation rates. The literature highlights the importance of inclusivity and responsiveness to diverse perspectives, dissenting voices and vulnerable groups who have the least capacity of exerting influence^31,38^. Engagement should transcend mere awareness-raising and give meaningful control to those engaged^34^ but still be “proportional to the nature and size of the PPP”^38^. New developments, such as those around AI-based analysis of data, should call for renewed PPI efforts^30,42^. Baric-Parker and Anderson^28^ suggest that decisions regarding the use of data by third parties should always involve input from patient representatives, and Cohen and Mello^63^ even argue that at least half of the members of hospital data access committees should be patients of that hospital. Such proposals can be seen as a response to the difficulties with individual informed consent. While inclusive public and patient engagement may be costly and takes time, it promotes public agency and trust in PPPs, improves the quality of stewardship and oversight, and ultimately helps achieve greater societal benefit by promoting the acceptability of the tools developed in PPPs^26,49,51^.

## Discussion

With this review, we have provided a broad overview of the ethical considerations involved in digital health research and development through public-private partnerships, and we hope this will help promote responsible innovation in PPPs. By addressing themes of data privacy and consent, public benefit and access, and good governance and trustworthiness, PPPs can better align with public expectations and safeguard societal values. From the literature, we synthesized fifteen key ethics recommendations across these themes, for PPPs to adopt Table 1. In addition to this overview of specific recommendations, our review has provided two general key findings that are relevant across all recommendations: (1) there is a lack of clarity on the meaning of ‘public benefit’ or ‘public interest’; (2) the discourse on ethics in PPPs has been mostly about the responsibilities of private entities, while public sector organizations should also (and even more so) be trustworthy and accountable. We elaborate on these two points in this Discussion.

However, before reflecting on these issues further, we should note the limitations of our review method and of the literature more generally. One issue is that most included articles discussed cases of PPPs in Europe, and also the more general articles were mostly written by authors from European and from English-speaking countries, so we call for further research assessing the ethical aspects of PPPs in other parts of the world. Another important limitation is that several included articles that we deemed to be inside our scope as the authors discussed ethical aspects of extensive exchanges between the public and private sector, did not actually use the term ‘PPP’. Sterckx et al.^37^ describe these as “concealed Public Private Initiative[s]” in which data collected by public organisations is shared with private partners without calling this a PPP. It seems that the term is mostly used explicitly in cases of highly formalised partnerships. Critical study on the concept ‘PPP’ is needed and should include normative analysis on what is a desirable form of partnership: we hypothesize that increased formalization is correlated with higher awareness of ethics, which may result in more equitable distribution of responsibilities and in increased public transparency. The nature and meaning of a PPP should also be challenged by exploring new forms of partnership that have genuine involvement of patients and publics, as we discuss hereafter.

The first general point for discussion is the criterion that digital health PPPs should benefit the public. While the governance of digital health PPPs should be based on questions of societal value and not just on data protection aspects, the reviewed literature shows that there is a lack of clarity and consensus on the meaning of ‘public benefit’ or the ‘public interest’, with different meanings used to justify PPPs. We find that PPPs need to be clear about what is meant with ‘public interest’ and patients and citizens should have a say in whether they want to contribute to this interest. Clarity on what constitutes public benefit or interest—including differentiation between these two overlapping terms—is important given the increasingly political aims of digital health research; evidenced in current ‘AI races’ between the US, China and the European Union, with the latter installing a European Health Data Space to promote medical AI development. It should first be clear who ‘the public’ is and if this includes merely patients or also the broader public. We think the latter would be suitable at least for large-scale PPPs with a large economic impact, or those that include data of healthy individuals. Wickson et al. argue that it is not productive nor democratic to think of ‘the public’ as a static entity, and that engagement should instead focus on the public as citizens: i.e., empowered individuals who are also members of communities^66^. They state that engagement should encourage those citizens to be active beyond the short, delineated time period of a project: “what is required is an informed citizenry empowered to actively engage in the democratic shaping of science and technology to meet social needs and accommodate plural values”. Similarly, Coeckelbergh argues for a “more active role of citizens and (end-)users: not only as participants in deliberation but also in ensuring, creatively and communicatively, that AI contributes to the common good”^67^. Thus, digital health PPPs should engage with various publics to find out what aims are acceptable and achieve clarity about what they understand to be a public benefit.

In this sense, we see a need for exploration of alternative partnership models such as ‘tripartite partnerships’ which add civil- or patient societies as equal and formal partners, as a a way to meaningfully incorporate public and patient values in PPPs. Tripartite partnerships were not described in the reviewed literature, while we feel the promotion of such partnerships might help balance out power somewhat in favour of patients and the general public. While the collaboration between the State of Iceland and deCODE Genetics has been called a tripartite partnership^45^, in that case citizens were not meaningful partners but merely shareholders if they bought stock in deCODE. We are not aware of published research on tripartite partnerships in digital health, but lessons may be learned from examples in the broader areas of healthcare^68^ and politics^69^. The World Health Organization has also recommended supporting mechanisms for community oversight of data that might be used in ‘data collectives’ or other partnerships^70^. Further work is needed on the practical, political and ethical questions related to setting up tripartite partnerships. In doing so, it is important to not simply ‘sync fast’ to keep up with innovation^71^, but rather to shape more sustainable innovation practices by taking the time to work on and demonstrate trustworthiness and to reflect on concerns together with stakeholders. Doing this successfully requires further work to explore effective incentives for stakeholders, what level of engagement is fair to expect from citizens, and how to avoid ‘participation fatigue’. It must be noted that even if appropriate engagement processes are in place, patients and citizens should not be pressured into ‘data solidarity’ for the common good but we find that they should remain able to (at least) opt out of the use of their data in PPPs.

Our second discussion point is about the responsibilities of public sector organizations participating in PPPs. Namely, our literature review showed that focusing only on the power of the private sector is too simplistic, as the interests of individual patients do not always align with governments’ or public sector organisations’ interests either (see the results section on ‘solidarization’). Important work has been done by authors like Shoshana Zuboff and Tamar Sharon who warn about ‘surveillance capitalism’^12^ and the ‘Googlization’ of health^22^, and we second these calls for more critical study of power asymmetries in PPPs. However, we find that framing the problem as Googlization has downsides. In particular, the rhetoric of big tech companies as (necessary) evil, which we saw in several articles in this review, may harm public trust in digital health PPPs. Especially when the private partner is a smaller company that collaborates on equal footing with a public organisation, this framing can be unfair and potentially harmful. Moreover, this frame might distract from the roles and responsibilities of public sector organizations whose role as data steward can conflict with the (implicit or explicit) aim of some digital health PPPs to contribute to broader economic development, rather than merely benefiting patients or the healthcare system.

Instead of ‘Googlization’, we think the broader concept of *‘economization of (digital) health’* is more fitting. Economization refers to a “gradual but sweeping takeover of all policy areas by concerns for the economy”^72^. Focusing on this broader trend as a useful frame studying the meaning of public benefit and how to achieve that in PPPs, would still allow the study of other ‘-zations’ like Googlization and monopolization while finally putting the spotlight on the motives of the public sector too. The discourse has been mostly about sharing publicly collected data with private entities, but vice versa, users of commercial digital devices might be reluctant to share their health data with governments. Rather than hide behind a frame of ‘Googlization’, government and academia should themselves be trustworthy and accountable, and they should take responsibility for governance and oversight in PPPs. When collaborating with private partners, stricter enforcement measures may be needed to counter some companies’ tendencies of ‘ethics washing’^73,74^. While beyond our scope to discuss here, lessons about accountability and trustworthiness can be learned from the existing literature base on trust in health research. Of note is that when we speak of trust in digital health PPPs, there is the question of not only *who* but also *what* data subjects are being asked to trust, e.g. the standards, training and codes regulating the field of digital health (or the lack thereof). We find that (inter-)national public sector organisations involved in the ethical, legal and social implications of medical research should develop general ethics guidelines for PPPs in the field of digital health, to help foster more successful partnerships that genuinely serve the public interest. The early and joint operationalisation of ethics would help digital health researchers to promote the public interest and to find a balance between respect for fundamental values and the pursuit of impactful innovation.

Development of general guidance for digital health PPPs could build on the study of existing (inter-)national biomedical ethics guidelines for recommendations about collaboration with industry, e.g. in the field of ‘Big Pharma’, to ascertain how these could apply to partnerships with ‘Big Tech’. Empirical research with diverse stakeholders could furthermore shed light on what constitutes the public interest, what expectations patients have, and how ethics committees are currently evaluating and overseeing PPPs. Such empirically informed and practice-oriented ethics guidance for digital health PPPs should be translated for each specific context of partnering – this translation might best be done by forming tripartite partnerships, as discussed in the previous section. In addition to anticipating the financial and legal viability of a PPP, co-developing ethics guidelines can “help reduce harm and prevent unjustified PPPs: defining an aligned vision, for instance, will likely reveal conflicts of interests – aligning on PPP guidelines early may thus serve as a litmus test for potential partners” (p. 657)^49^. If PPPs would become more acceptable and successful as a result of the development of ethics guidelines, this also contributes to reducing the research waste of failed collaborations.

## Methods

### Search strategy and inclusion criteria

Studies were identified by searching three databases: PubMed, EMBASE and Web of Science (Supplementary Table 3: search queries). Our systematic search included records published in English, in the last decade leading up to 1 December 2023. Free search terms and subject headings (MeSH and Emtree) were selected that were related or synonymous to: ‘public-private partnerships’, ‘ethics’, ‘digital health’. Theoretical and empirical studies that critically reviewed the ethical aspects of PPPs within digital health were included in this scoping review. We included studies that discussed ‘data partnerships’, ‘strategic partnerships’, or both. Articles were excluded that merely mentioned ethical aspects of PPPs in digital health and provided no argumentation.

### Study screening and data extraction

Titles and abstracts were first screened by one of the authors (DH). Articles that did not contain the key concepts of this review were excluded. If the author was unsure of whether an article aligned with the criteria, the article was discussed with another author (MB). Second, full-text records were independently evaluated by both authors (DH and MB) using the online COVIDENCE tool (www.covidence.org) for literature screening, after which disagreements were resolved by discussion. Reference lists of the included articles were searched manually to identify additional relevant publications which were added to the pool of selected studies (backward reference searching). Also, citations of the included records by other articles were used to find additional records (forward reference searching).

### Analysis and reporting

After screening was concluded, DH and MB carefully read all included papers and highlighted (‘coded’) important findings. Analysis followed the method of qualitative content analysis^75^ and was an iterative process of moving between inductive coding to identify key themes, and deductive coding to categorise findings according to these themes. The resulting code structure was discussed among all authors and noted down in an extraction table which served as the basis for the narrative description in this paper. Finally, the main themes were linked to the two major phases of digital health research: the collection and sharing of data to develop digital health tools, followed by the implementation and use of these tools (cf^28^). In reporting our findings, this review followed PRISMA scoping review guidelines (PRISMA-ScR)^76^ (Supplementary Table 4: PRISMA-ScR checklist).

## Supplementary information

Supplementary Information

## Data Availability

No datasets were generated or analysed during the current study.
