# Supplementary material for: A scoping review of ethical aspects of public-private partnerships in digital health
Source: NPJ Digit Med. 2025 Feb 27;8:129. doi: 10.1038/s41746-025-01515-3 (PMC11868512; doi:10.1038/s41746-025-01515-3)
Supplement: Supplementary file 1 — Supplementary Information [file 41746_2025_1515_MOESM1_ESM.pdf]

SUPPLEMENTARY TABLE 1 – PRISMA flowchart for screening and inclusion

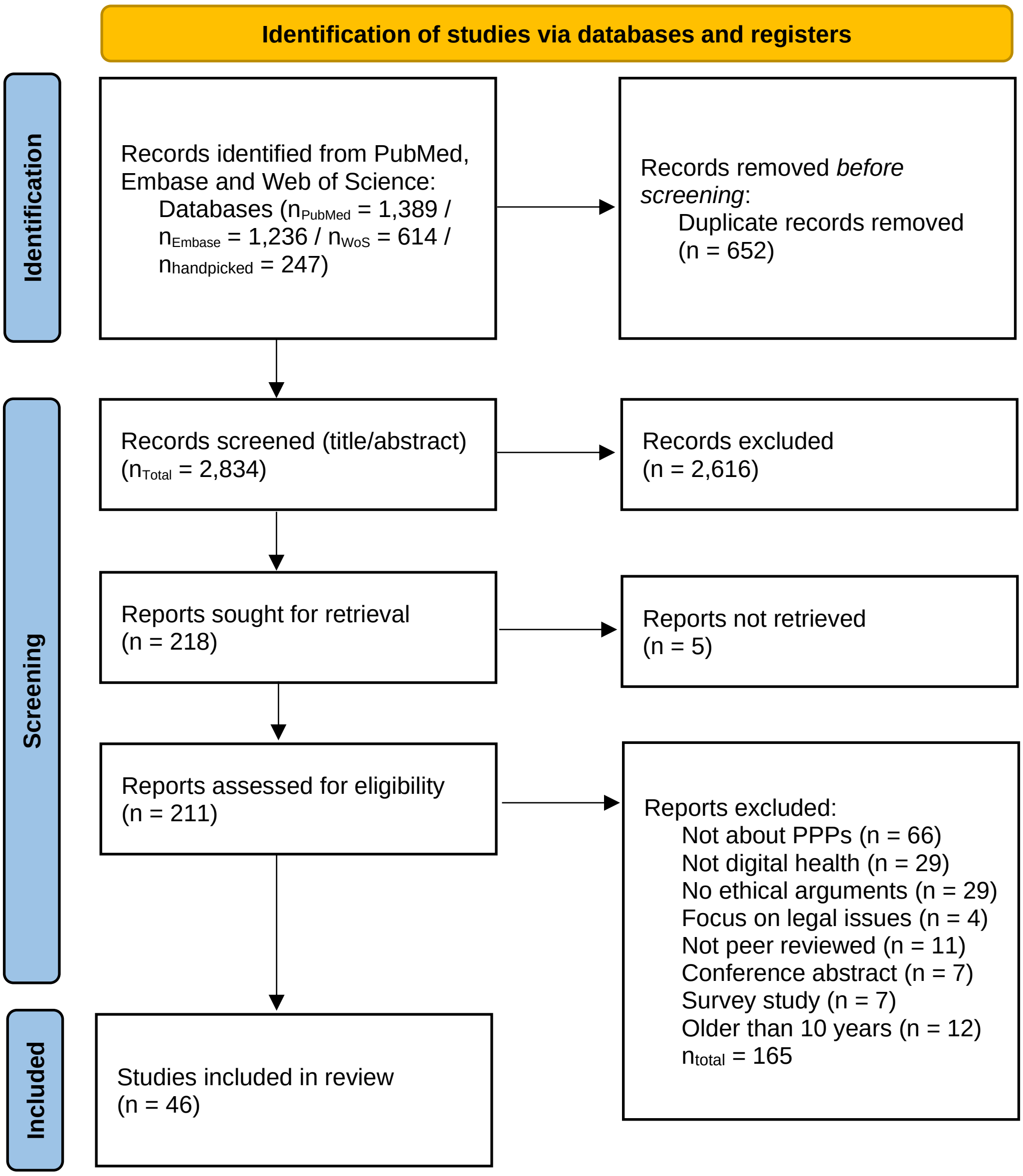

SUPPLEMENTARY TABLE 2 – Characteristics of included studies in the review

Name of first author	Reference year	Country*	Context	Cases discussed	Aim of the paper	Conclusion
Arnason & Andersen	2013	Iceland	Genetics	deCODE	To explain the history of deCODE Genetics Inc. and how this corporation was permitted to set up a controversial health and genetic database of Icelandic people.	It was found that the first controversial database was not set up due to the law allowing this database was unconstitutional. Instead, deCODE created a different database collection of genome-wide information through its studies. However, this database was a commercial failure and went bankrupt in 2009, being bought out by Amgen in 2012.
Ballantyne & Stewart	2019	UK	Health data for precision medicine	NHS/DeepMind	To discuss three specific, ethical challenges for PPPs by applying a deliberative framework.	Using the deliberative framework allows to articulate different ethical trade-offs in PPPs such as appropriate data-sharing, values and helps support deliberation and communicating reasoning to stakeholders.
Ballantyne et al.	2022	Singapore	Health data	National Precision Medicine Initiative	To provide informed views from a citizens' jury in Singapore on whether sharing precision medicine data with private industry would be permissible, and if so, under which circumstances.	The jury concluded that sharing precision medicine data should be permissible under a set of nine conditions, which aligned with previous studies on data sharing with private industry.
Baric-Parker & Anderson	2020	USA	Health data for Artificial Intelligence (AI)	General	To create and present recommendations for the ethical and social challenges in electronic health record (EHR) data-sharing collaborations with AI developers in light of the Rome Call to AI ethics (RCAIE) document and the guiding principles of the Ethical and Religious Directives for Health Care Services (ERDs).	The existing regulatory guidance in PPPs has not kept pace with the new technological advances and the ERDs lack specificity. The RCAIE fills a critical gap in knowledge. However, more collaboration between the church and private parties is required to close the gap as the field swiftly develops.

Carter et al.	2015	UK	Health data	care.data	To show how the concept of a social licence can explain the challenges within the care.data program and whether it failed to secure a social licence.	It was proposed that the care.data program failed to obtain a social licence because of three issues underlying the care.data program, namely due to problems in warrants of trust, an implied rupture in the traditional role, and the uncertainty on whether care.data was a public benefit.
Cheung	2020	UK	Health data	General	To focus on the key roles that public benefit plays in getting access to the UK's health database by using the concept of 'trade-off fallacy" to argue that current data access largely negates the possibility of control by individuals regarding future use of their personal health data.	The article suggests that although public health data is protected through robust legal frameworks like the GDPR, it is still likely to be subjected to similar conditions of data gathering elsewhere in the digital economy where the private industry is involved with the inability of individuals to control their data.
Cohen & Mello	2018	USA	Health data	Dinerstein v. Google	To highlight the issues of the current HIPAA act in the United States of America by using the Dinerstein v. Google court case.	The Dinerstein v. Google case suggests that the US health data regulatory regime through the HIPAA act is outdated and that improvements surrounding data sharing governance are necessary.
Cole et al.	2020	USA	Health data	General	To develop ten key principles after discussions with various stakeholders at multiple institutions to guide responsible use and sharing of clinical data for research and care.	The ten principles that were created can be used by both universities and healthcare providers to build upon. Moreover, these principles may provide a chance of holding faculty staff, leaders and business partners accountable.
de Lecuona & Villalobos-Quesada	2018	EU	Health data and biobanks	Catalan VISC+/PADRIS; UK Biobank	To enquire about the value of personal (health) data and its proper use by analysing two EU cases as perspectives.	The public and private sector participate with different goals in mind which might conflict with fundamental rights, most notably privacy. The authors advocate for stopping the commercialisation and unjustified gathering of data to protect the interests of citizens.

Ferretti & Vayena	2022	General (Italy*)	Health data	COVID-19 digital epidemiology	To leverage PPP examples from the COVID-19 pandemic to investigate ethical issues that were overlooked and their implications, by discussing three themes: i. the digital divide; the role of technology companies; re-use of personal health data.	The COVID-19 pandemic has shown that data used for epidemic surveillance must be protected. Without proper engagement on the three issues discussed, digital epidemiology tools may undermine equity and public trust, whereas a broader governance approach could enhance their effectiveness in combating epidemics.
Fisher & Rosenhek	2022	Israel	Health data	Israeli National Plan for Digital Health	To analyse the Israeli National Plan for Digital Health as a socio-technical assemblage.	It was uncovered that the creation of new digital health assemblages is never purely emergent nor completely dehierarchized and decentered. Furthermore, the authors highlight the importance of specific imaginaries of digital health data in the process of forming these assemblages.
Graham	2021	General (UK*)	Health data	General	To examine if people's interactions concerning health data sharing with commercial companies should be based on trust, reliance or confidence.	Appropriate health data usage by commercial companies should be based on confidence rather than trust in the company. However, there might still be a role for trust in a framework for sharing health data.
Grön	2021	Finland	Health data	The Apotti healthcare information system renewal project	To understand how the development of Apotti has involved different stakeholders to create collective expectations, and to analyse these using the sociology of expectations and justification theory to identify the common good between the stakeholders.	It is identified that the ambiguity and plurality of the common good in data-intensive healthcare raises concerns on how it can shape healthcare in the future and that the plural understandings of the common good might conflict with each other.

Holm & Ploug	2017	Denmark	Health data	General	To examine the Danish model of health data sharing and to investigate which of four different models of informed consent would protect citizens as data subjects in the Danish system if private parties were to get involved.	In the current Danish system, health data governance should be improved by requiring increased involvement of research ethics committees (RECs), better training of researchers in research ethics and implementing a meta-consent model.
Horn & Kerasidou	2020	UK	Health data	General	To explore how PPPs between the NHS and private parties change the solidaristic character of the NHS and impact public trust. Also, ways to maintain public trust are shown by creating a partnership model with companies seeking access to patient data based on solidarity.	By engaging in partnerships with profit-oriented companies, the trust in the NHS is tested. To maintain this trust, solidarity-based partnerships that serve collective interests are needed. If trust is not maintained, data-driven health care by the NHS will not be reliable.
Howard	2021	General (Australia*)	Wearable devices in healthcare	General	To argue that foundational assumptions about efficiency, autonomy and neoliberal policies in healthcare are leading to a devaluation of equity and cannot meet the particular needs of people and groups.	The pervasive influence of techno-optimism and solutionism, driven by technology corporations, pose a significant threat to traditional institutions and equity in healthcare delivery. When developing new mHealth interventions and wearable devices, the risks of overlooking diversity and reinforcing normative constructs should be acknowledged.
Kaplan	2015	UK and US	Health data	Court cases of Sorrell and Source	To analyse how health data privacy can be impacted if corporations sell data, using two court cases, and recommending policy changes for better privacy protection.	If data collected for one purpose is used for another purpose (e.g. marketing), public confidence can be undermined, especially if the general public is unaware of this secondary use. The court cases raise ethical questions about this commodification of medical information and the harmonisation of policy across jurisdictional boundaries.

Kaplan	2016	UK and US	Health data	Court cases of Sorrell and Source	To analyse two court cases about selling prescription data that raised questions on the meaning of privacy and public interest, as well as additional ethical issues (e.g. drug detailing, clinician privacy etc.).	New technological and data sharing developments require revisiting policies that fail to protect the privacy of subjects. As one of the courts noted: “The capacity of technology to find and publish personal information … present serious and unresolved issues concerning personal privacy and the dignity it seeks to secure.” (*Sorell v. IMS Health Inc. et al., 2011)*
Landers et al.	2023	Switzerland	Health data	Swiss Personalized Health Network	To report the development of creating the Swiss Personalized Health Network’s ethical guidelines and to describe the underlying ethical issues of this process and how stakeholders are incorporated to address these issues.	The paper highlights several strategies for overcoming these ethical issues in the context of Swiss health data. Namely, this by agreeing and formalizing ethical principles and practices at the beginning of a PPP, as both partners and society can benefit from the relationship when there is a mutual commitment to ethical principles.
Lanzing et al.	2022	EU	Contact tracing apps	Google/Apple and EU in the pandemic	To investigate how the dependency frame of the EU on Big Tech cannot fully capture the involvement and development of ‘Gapple’ infrastructure for the EU contact tracing apps, by proposing a novel approach that builds on the concept of co-production.	The authors argue that the relationship between EU countries and Big Tech in this context is not one of simple dependency but a complex, mutual shaping of policy and technology. This "techno-tango" highlights shifting power dynamics, where both parties alternately lead, contributing to the debate on public-private partnerships in digital health governance.
Laurie	2019	General (UK*)	Health data and wearables	Hypothetical case of wearable technology foundation Veteris	To consider the issues and challenges that arise within cross-sectoral sharing of data by using the deliberative framework of Xafis et al. (2019) and applying it to a hypothetical case study.	Application of the framework demonstrates that the need for ethical reflection and action is central to obtaining any form of success in initiatives that try to tackle cross-sectoral sharing of data.

Lipworth	2019	General (Australia*)	Health data	Hypothetical case of sharing electronic medical records	To apply an ethical framework to the use of real-world data (RWD) for assessing health interventions, addressing issues such as data quality and the influence of commercial interests, by applying the deliberative framework of Xafis et al. (2019).	Using RWD is not always viable since it is often derived from secondary sources, the size of the datasets exacerbates methodological problems and because a lack of experience in RWD renders it susceptible to political, commercial and professional biases.
Marelli et al.	2021	EU	Health data	General	To evaluate how the GDPR affects health research by reviewing the policy trajectory of the GDPR, examining potential challenges elicited by Big Tech in the healthcare domain, and proposing a use-based data governance framework.	As it stands, Big Tech elicit problems in the health domain such as privatization, enclosure and market dominance, which the GDPR is not able to address within the regime for research; a re-purposing of data protection and data governance is required.
Martin & Hollin	2014	UK	Health data and biobanks	UK Biobank, Genomics England, care.data	To study the ethical, social, and policy implications of changes in the relationship between the public and private sector, by focussing on three large-scale UK projects.	Despite changes within the digital health framework, such as the increasing justification of extracting private value from public data, it is suggested that there has been no fundamental change in the direct engagement of the industry with the public sector in the area of health data. However, it is apparent that the public sector itself has increasingly changed to align with business methods and the industry's needs.
Piciocchi et al.	2018	Italy	Health and genetic data	SharDNA and the Christ project	To outline challenges faced by the Italian legal system on the regulation of public-private genetic biobanks, especially during biobank insolvencies.	Two case studies demonstrate how a civil-law country like Italy managed the ethical, legal, and societal implications of biobank regulation and governance. Both cases show the need to develop cooperation and synergies between public and private actors to manage these biobanks and to conciliate patent law and public interest.

Powles & Hodson	2017	UK	Health data for AI	DeepMind/NHS Royal Free	To analyse and draw lessons from the DeepMind-Royal Free case on the sharing of population-derived datasets to large private companies by identifying critical questions for industry, policy-makers and individuals.	It is imperative that institutions like the NHS explore innovative ways to advance their health mission, and AI offers great promise. However, the PPP between DeepMind and Royal Free did not deliver on this promise due to many ethical issues that arose during development.
Powles & Hodson	2018	UK	Health data for AI	DeepMind/ NHS Royal Free	To respond to a 47-point letter by DeepMind, which in turn was a response to the authors' 2017 case study of the collaboration between DeepMind and the NHS Royal Free Hospital Trust.	In this response, the authors clarify that their critique centres on the lack of a valid legal basis and inadequate safeguards for handling patient data in this PPP. They emphasize that technology's potential should not override established principles.
Rantanen & Heimo	2018	Finland	Health data	Finnish government project for sharing EHR data	To review the problem of selling or sharing citizen's health data for secondary use to other organisations, by reflecting the Finnish system throughout the article.	Pitfalls in privatising health data are identified, and it is argued that even when data are anonymised before selling or sharing, it may still be identifiable; full anonymisation without losing the value of the data, is deemed impossible.
Schneider	2022	General (Italy*)	Health data	Deepmind/NHS Royal Free Hospital; IBM/Lombardia Region	To investigate the challenges of how digital health data is collected by private and public institutions, and the complexity of interests and risks stemming from health data pooling practices.	Regulatory measures must balance the benefits of health data sharing with the protection of privacy, data rights, and the prevention of monopolies in digital health markets.
Shah et al.	2021	England, Iceland and Sweden	Health data	Scenarios bases on key cases from the three countries	To understand citizens' views on the acceptability of health data use in different contexts, ranging from healthcare to research, and to commerce and marketing.	Citizens' views centred on four points: data should be ethically used, even when there is commercial interest; subjects and public institutions should receive benefits; third parties' data use requires more transparency and accountability; better information should be given to empower data subjects.

Sharon	2016	General (the Netherlands*)	Health research	General	To examine the "Googlization" of health research and the ethical issues that arise from this.	Three ethical concerns arise: power asymmetries, quality of research, and privacy/informed consent. These issues are underpinned by different appeals to different values and create tensions, such as the discriminatory effects of big data and its perceived objectivity, open versus proprietary data sources, public versus private interests and participatory science versus new power balances.
Sharon	2018	General (the Netherlands*)	Health research	General	To identify the different moral repertoires and conceptualizations of the common good that are present in the Googlization of health research.	Current studies of public-private data sharing tend to frame it in terms of public benefit versus private financial gains, but this paper shows that there are multiple conceptions of the common good at stake.
Sharon	2020	General (the Netherlands*)	Contact tracing apps	General	To argue that Gapple's API is encroaching into new spheres of our social life, where the companies gain (through legitimate advantages within digital expertise) illegitimate access to the spheres of health, medicine, and politics.	While privacy concerns dominate the debate on digital contact tracing, the article argues that the broader risks of tech corporations gaining influence in public sectors like healthcare and policy-making are being overlooked. This growing influence may result in dependencies on private actors for public goods and the accumulation of decision-making power across various societal spheres.
Snell et al.	2021	Nordic welfare states/Finland	Health data	Findata	The article explores the tension between Nordic welfare state values of solidarity and the emerging data-driven health economy, focusing on how health data is repurposed for economic gain.	While the data-driven health economy depends on the welfare state's data regime, it contradicts the solidarity-based values that justify data collection, thus creating a solidarity paradox. The concept of "solidarization" is introduced to highlight how individuals are expected to support these processes despite conflicting interests.

Spector-Bagdady et al.	2020	USA	Health data and biospecimens	Michigan Medicine's approach	To show how Michigan Medicine applies a principlist approach to using deidentified data and to codify a review and authorization protocol for sharing data and biospecimens with external entities.	Several challenges were faced during the creation of a standard protocol for researchers, such as data related to rare diseases in which participants can no longer be contacted for reconsent, secondary access by commercial companies, blurry lines between clinical services, quality improvement and research, and consent for broad reuse with no information about the specific companies involved.
Spithoff et al.	2022	Canada	Health data brokers	General	To provide insights on usage of deidentified Canadian patient data by commercial data brokers; discussing potential harms, public opinion about the use of their data and how to strengthen existing policies.	A coordinated infrastructure for the use of health data across Canada and an updated privacy legislation would protect citizens and enable appropriate data use.
Sterckx et al.	2016	UK	Health data	care.data	To provide insights into concerns identified by citizens on the care.data scheme, by analysing readers' comments on published news articles and on the official NHS care.data website.	Most notably present amongst these comments of citizens was their wish to further the public benefit without being manipulated into doing it, while at the same time being protected against abuses.
Sterckx et al.	2018	UK	Health data and genomics	care.data; Genomics England	To consider the ethical concerns surrounding the collection of health-related data in the care.data 2.0 and 100 k GP projects in the UK regarding privacy, autonomy and justice.	Both cases use the reputation of the NHS to appear trustworthy, but this trust should be merited and not manufactured. Transparency on how the involvement of industry affects the nature and extent of the benefits to society, is a prerequisite for both trust and trustworthiness.

Street et al.	2021	Australia	Health data	General	To provide informed views from two Australian citizens' juries on sharing health data with the private industry for development and research.	Both juries supported the sharing of health data with the private industry for development and research as long as certain conditions were in place (such as penalties for misuse, oversight by an independent body, and requirements for the release of information about the use of the health data by the private party).
Tully et al.	2019	UK	Health data	Hypothetical cases of NHS data sharing	To examine what informed citizens considered to be the appropriate use of health data within in learning health system.	Citizens tended to be more accepting of sharing data with both private and public partners after the citizens' jury, and jurors accepted commercial gain if public benefit was achieved as well.
Tupasela	2021	Iceland	Genetics	deCODE	To provide an overview of different cases where people's health data is turned into an asset by both companies and states.	The study reveals that population branding, particularly in Denmark and Finland, has become a tool for marketing national biobank data and healthcare resources to attract investment, raising concerns about the commodification of populations and the ethical implications of transforming health data into a commercial product.
Vezyridis & Timmons	2017	UK	Health data	care.data	To study the controversies surrounding the care.data program by using Nissenbaum's framework of privacy as contextual integrity and to describe the data flows surrounding care.data.	The analysis argues that the care.data programme prioritizes economic and scientific goals over social and ethical considerations, reducing privacy to an individual concern. It suggests that unless these broader values are addressed, such healthcare data-sharing initiatives will face continued resistance.

Winickoff	2015	Iceland	Genetics	deCODE	To explain the history of the deCODE biobank and the Icelandic Health Sector Database Act in detail and to examine ethical flaws during development of this nationwide project.	The deCODE case sparked a debate about science, technology, and nationhood. Supporters saw it as key to economic growth, while critics believed it compromised democratic values and scientific ethics. Both sides agreed that science and technology play a crucial role in shaping national identity, reflecting global tensions in balancing innovation with ethical concerns.
Winkler et al.	2023	General (Germany*)	Health data	General	To advance the debate on the use of medical data generated by the public sector for use in the private sector.	Use of health data by for-profit companies should be granted if they meet certain conditions, namely that their actions need to be in the public interest and that they respect patients' informational rights.
Winter & Davidson	2019	UK	Health data	DeepMind / NHS Royal Free	To investigate how forms of data governance were adapted in the case of the DeepMind / Royal Free partnership, to be able to address concerns of contextual integrity of personal health information.	To be able to effectively govern the increasingly more networked nature of personal health information, it is necessary to examine each context where this data is generated, (re)used, and who the stakeholders are, including their relevant values and interests.

Witjas-Paalberends et al.	2023	The Netherlands	Data-driven healthcare	General	To elucidate (ethical) challenges in managing big data-driven health care innovations by PPPs in the Netherlands and how to overcome them.	The study highlights key challenges like data variety, quality, and sharing, with PPPs facing additional hurdles such as conservatism toward data-driven decision-making. It recommends a combination of traditional business intelligence methods, fostering collaboration, and building trust between stakeholders as essential steps to fully harness big data's potential in healthcare innovations.

* In instances of a general setting, the country where the first author originates from is mentioned.

SUPPLEMENTARY TABLE 3 – Search queries for PubMed, Web of Science and EMBASE

PubMed

Initial search performed on 7 February 2023:

#1 Digital health	"medical records systems, computerized"[MeSH] OR "medical informatics"[MeSH] OR "telemedicine"[MeSH] OR "digital technology"[MeSH] OR "health care sector"[MeSH] OR "eHealth"[tiab] OR "e-Health"[tiab] OR "m-Health"[tiab] OR "digital health"[tiab] OR "health technolog*"[tiab] OR "mHealth"[tiab] OR "data-intensive medicine"[tiab] OR "data intensive medicine"[tiab] OR "data-driven medicine"[tiab] OR "data-driven medicine"[tiab] OR "health informatics"[tiab] OR "big data"[tiab] OR "health data"[tiab] OR "digital capitalism"[tiab] OR "health apps"[tiab] OR "electronic health record*"[tiab] OR "EHR"[tiab] OR "health data*"[tiab] OR "robot*" [tiab] OR "wearable*"[tiab]	695,677
#2 Public-private partnerships	"public private partnership*"[tiab] OR "PPP"[tiab] OR "PPPs"[tiab] OR "public private partnership*"[tiab] OR "academic industry collaboration*"[tiab] OR "academic industry collaboration*"[tiab] OR "intersectoral collaboration*"[tiab] OR "intersectoral collaboration"[MeSH] OR "AIC"[tiab] OR "AICs"[tiab] OR "public private sector*"[tiab] OR "public private sector*"[tiab] OR "commerc*"[tiab] OR "commerce"[MeSH] OR "private sector"[tiab] OR "public sector"[tiab] OR "commod*"[tiab] OR "valorisation"[tiab] OR "valorization"[tiab] OR "corporation*"[tiab] OR "compan*"[tiab] OR "industries"[tiab] OR "industry"[tiab] OR "capitalis*"[tiab]	687,354
#3 Ethics	"bioethic*"[tiab] OR "ethic*"[tiab] OR "ethics"[MeSH] OR "moral*"[tiab] or "consent"[tiab]	341,076
#4 Languages	(English[Language] or Dutch[Language])	
Results	#1 AND #2 AND #3 AND #4	1,300

The search was updated on 28th of November, 2023:

#5 Filter	(“2023/02/08”[Date-Publication] : “3000”[Date-Publication])	
Results	#1 AND #2 AND #3 AND #4 AND #5	88
**Total**		**1,388**

Web of Science

Initial search performed on 7 February 2023:

#1 Digital health	(TI=(“ehealth”) OR TI=(“e-health”) OR TI=(“mhealth”) OR TI=(“m-health”) OR TI=(“digital health”) OR TI=(“health technolog*”) OR TI=(“data-intensive medicine”) OR TI=(“data intensive medicine”) OR TI=(“data-driven medicine”) OR TI=(“data driven medicine”) OR TI=(“health informatics”) OR TI=(“big data”) OR TI=(“health data*”) OR TI=(“digital	323,858

	capitalism”) OR TI=(“health apps”) OR TI=(“electronic health record*”) OR TI=(“EHR”) OR TI=(“robot*”) OR TI=(“wearable*”) OR AB=(“ehealth”) OR AB=(“e-health”) OR AB=(“mhealth”) OR AB=(“m-health”) OR AB=(“digital health”) OR AB=(“health technolog*”) OR AB=(“data-intensive medicine”) OR AB=(“data intensive medicine”) OR AB=(“data-driven medicine”) OR AB=(“data driven medicine”) OR AB=(“health informatics”) OR AB=(“big data”) OR AB=(“health data*”) OR AB=(“digital capitalism”) OR AB=(“health apps”) OR AB=(“electronic health record*”) OR AB=(“EHR”) OR AB=(“robot*”) OR AB=(“wearable*”))	
#2 Public-private partnerships	(TI=(“public private partnership”) OR TI=(“PPP”) OR TI=(“PPPs”) OR TI=(“public-private partnership”) OR TI=(“academic industry collaboration”) OR TI=(“academic-industry collaboration”) OR TI=(“AIC”) OR TI=(“AICs”) OR TI=(“intersectoral collaboration”) OR TI=(“public private sector”) OR TI=(“public-private sector”) OR TI=(“commerc*”) OR TI=(“private sector”) OR TI=(“public sector”) OR TI=(“valorisation”) OR TI=(“valorization”) OR TI=(“corporation”) OR TI=(“company”) OR TI=(“companies”) OR TI=(“industry”) OR TI=(“industries”) OR TI=(“capitalis*”) OR TI=(“commod*”) OR AB=(“public private partnership”) OR AB=(“PPP”) OR AB=(“PPPs”) OR AB=(“public-private partnership”) OR AB=(“academic industry collaboration”) OR AB=(“academic-industry collaboration”) OR AB=(“AIC”) OR AB=(“AICs”) OR AB=(“intersectoral collaboration”) OR AB=(“public private sector”) OR AB=(“public-private sector”) OR AB=(“commerc*”) OR AB=(“private sector”) OR AB=(“public sector”) OR AB=(“valorisation”) OR AB=(“valorization”) OR AB=(“corporation”) OR AB=(“company”) OR AB=(“companies”) OR AB=(“industry”) OR AB=(“industries”) OR AB=(“capitalis*”) OR AB=(“commod*”))	1,973,891
#3 Ethics	(TI=("bioethic*") OR TI=("ethic*") OR TI=("moral*") OR TI=("consent") OR AB=("bioethic*") OR AB=("ethic*") OR AB=("moral*") OR AB=("consent"))	488.239
#4 Language	LA=(English OR Dutch)	
Results:	#1 & #2 & #3 & #4	532

The search was updated on 28th of November, 2023:

#5 Filter	Not a query, but a filter on WoS: 08-02-2023 until 01-01-3000	
Results	#1 AND #2 AND #3 AND #4 AND #5	80
**Total**		**612**

EMBASE

Initial search performed on 7 February 2023:

#1 Digital health	(‘electronic medical record system’)/exp OR (‘medical informatics’)/exp OR (‘telemedicine’)/exp OR (‘digital technology’)/exp OR (‘health care industry’)/exp OR ((‘eHealth’):ti,ab) OR ((‘e-Health’:ti,ab)) OR ((‘m-Health’):ti,ab) OR ((‘digital health’):ti,ab) OR ((‘health technolog*’):ti,ab)	322,940

	OR ((‘mHealth’):ti,ab) OR ((‘data-intensive medicine’):ti,ab) OR ((‘data intensive medicine’):ti,ab) OR ((‘data-driven medicine’):ti,ab) OR ((‘data-driven medicine’):ti,ab) OR ((‘health informatics’):ti,ab) OR ((‘big data’):ti,ab) OR ((‘health data’):ti,ab) OR ((‘digital capitalism’):ti,ab) OR ((‘health apps’):ti,ab) OR ((‘electronic health record*’):ti,ab) OR ((‘EHR’):ti,ab) OR ((‘health data*’):ti,ab) OR ((‘robot*’):ti,ab) OR ((‘wearable*’):ti,ab)	
#2 Public-private partnerships	((‘public private partnership*’):ti,ab) OR ((‘PPP’):ti,ab) OR ((‘PPPs’):ti,ab) OR ((‘public private partnership*’):ti,ab) OR (‘Public-private partnership’)/exp OR ((‘academic industry collaboration*’):ti,ab) OR ((‘academic industry collaboration*’):ti,ab) OR ((‘intersectoral collaboration*’):ti,ab) OR (‘intersectoral collaboration’)/exp OR ((‘AIC’):ti,ab) OR ((‘AICs’):ti,ab) OR ((‘public private sector*’):ti,ab) OR ((‘public private sector*’):ti,ab) OR ((‘commerc*’):ti,ab) OR (‘commercial phenomena’)/exp OR ((‘private sector’):ti,ab) OR ((‘public sector’):ti,ab) OR ((‘commod*’):ti,ab) OR ((‘valorisation’):ti,ab) OR ((‘valorization’):ti,ab) OR ((‘corporation*’):ti,ab) OR ((‘compan*’):ti,ab) OR ((‘industries’):ti,ab) OR ((‘industry’):ti,ab) OR ((‘capitalis*’):ti,ab)	951,616
#3 Ethics	((‘bioethic*’):ti,ab) OR ((‘ethic*’):ti,ab) OR (‘ethics’)/exp OR ((‘moral*’):ti,ab) or ((‘consent’):ti,ab)	549,099
#4 Language	[Dutch]/lim OR [English]/lim	
Results:	#1 & #2 & #3 & #4	1,135

An extra search was carried out on 28th of November, 2023

#5 Filter	[2023-2024]/py AND [08-02-2023]/sd	
Results	#1 AND #2 AND #3 AND #4 AND #5	100
**Total**		**1,235**

SUPPLEMENTARY TABLE 4 – Preferred Reporting Items for Systematic reviews and Meta-Analyses extension for Scoping Reviews (PRISMA-ScR) Checklist

SECTION	ITEM	PRISMA-ScR CHECKLIST ITEM	REPORTED IN SECTION
**TITLE**			
Title	1	Identify the report as a scoping review.	Title
**ABSTRACT**			
Structured summary	2	Provide a structured summary that includes (as applicable): background, objectives, eligibility criteria, sources of evidence, charting methods, results, and conclusions that relate to the review questions and objectives.	Unstructured abstract as per journal formatting requirements
**INTRODUCTION**			
Rationale	3	Describe the rationale for the review in the context of what is already known. Explain why the review questions/objectives lend themselves to a scoping review approach.	Reviewing ethical aspects is necessarily done as a scoping review as no meta-analysis can be done.
Objectives	4	Provide an explicit statement of the questions and objectives being addressed with reference to their key elements (e.g., population or participants, concepts, and context) or other relevant key elements used to conceptualize the review questions and/or objectives.	Introduction
**METHODS**			
Protocol and registration	5	Indicate whether a review protocol exists; state if and where it can be accessed (e.g., a Web address); and if available, provide registration information, including the registration number.	N/A
Eligibility criteria	6	Specify characteristics of the sources of evidence used as eligibility criteria (e.g., years considered, language, and publication status), and provide a rationale.	Methods
Information sources*	7	Describe all information sources in the search (e.g., databases with dates of coverage and contact with authors to identify additional sources), as well as the date the most recent search was executed.	Methods
Search	8	Present the full electronic search strategy for at least 1 database, including any limits used, such that it could be repeated.	Supplementary Table 3
Selection of sources of evidence†	9	State the process for selecting sources of evidence (i.e., screening and eligibility) included in the scoping review.	Methods
Data charting process‡	10	Describe the methods of charting data from the included sources of evidence (e.g., calibrated forms or forms that have been tested by the team before their use, and whether data charting was done independently or in duplicate) and any processes for obtaining and confirming data from investigators.	Methods
Data items	11	List and define all variables for which data were sought and any assumptions and simplifications made.	Methods
Critical appraisal of individual	12	If done, provide a rationale for conducting a critical appraisal of included sources of evidence;	N/A

SECTION	ITEM	PRISMA-ScR CHECKLIST ITEM	REPORTED IN SECTION
sources of evidence§		describe the methods used and how this information was used in any data synthesis (if appropriate).	
Synthesis of results	13	Describe the methods of handling and summarizing the data that were charted.	Methods
**RESULTS**			
Selection of sources of evidence	14	Give numbers of sources of evidence screened, assessed for eligibility, and included in the review, with reasons for exclusions at each stage, ideally using a flow diagram.	Supplementary Table 1
Characteristics of sources of evidence	15	For each source of evidence, present characteristics for which data were charted and provide the citations.	Supplementary Table 2
Critical appraisal within sources of evidence	16	If done, present data on critical appraisal of included sources of evidence (see item 12).	N/A
Results of individual sources of evidence	17	For each included source of evidence, present the relevant data that were charted that relate to the review questions and objectives.	Available upon request
Synthesis of results	18	Summarize and/or present the charting results as they relate to the review questions and objectives.	Results
**DISCUSSION**			
Summary of evidence	19	Summarize the main results (including an overview of concepts, themes, and types of evidence available), link to the review questions and objectives, and consider the relevance to key groups.	Discussion
Limitations	20	Discuss the limitations of the scoping review process.	Discussion
Conclusions	21	Provide a general interpretation of the results with respect to the review questions and objectives, as well as potential implications and/or next steps.	Discussion
**FUNDING**			
Funding	22	Describe sources of funding for the included sources of evidence, as well as sources of funding for the scoping review. Describe the role of the funders of the scoping review.	N/A
